# 非小细胞肺癌组织miRNA-221表达及其与预后的关系研究

**DOI:** 10.3779/j.issn.1009-3419.2014.03.07

**Published:** 2014-03-20

**Authors:** 俊杰 吕, 磊 徐, 有涛 许, 满堂 邱, 欣 杨, 洁 王, 荣 尹, 林 许

**Affiliations:** 1 210009 南京，南京医科大学附属江苏省肿瘤医院胸外科，江苏省恶性肿瘤分子生物学及转化医学重点实验室 Department of Thoracic Surgery, Jiangsu Key Laboratory of Molecular and Translational Cancer Research, Nanjing Medical University Affiliated Cancer Hospital of Jiangsu Province, Nanjing 210009, China; 2 223300 淮安，淮安市第一人民医院胸外科 Department of Thoracic Surgery, Huai'an First People's Hospital, Nanjing Medical University, Huai'an 223300, China

**Keywords:** miRNA-221, 肺肿瘤, 基因表达, 预后, miRNA-221, Lung neoplasms, Gene expression, Prognosis

## Abstract

**背景与目的:**

microRNAs是肿瘤的重要调控因子，其中miRNA-221的表达与多种肿瘤相关，本研究旨在探讨miRNA-221在非小细胞肺癌组织中的表达及其与预后的关系。

**方法:**

分析江苏省肿瘤医院2005年11月-2007年1月期间行手术治疗的117例非小细胞肺癌患者的临床资料。采用实时定量逆转录聚合酶链反应检测上述病例肺癌标本中miRNA-221的水平；应用*χ*^2^检验分析miRNA-221表达水平与各临床病理参数的关系；采用*Kaplan-Meier*方法分析miRNA-221表达与非小细胞肺癌生存期（overall survival, OS）之间的关系；采用*Cox*风险比例模型分析各个协变量的联合效应。以*P* < 0.05认为差异有统计学意义。

**结果:**

miRNA-221的表达量与患者年龄相关，与患者性别（*χ*^2^=0.070, *P*=0.791）、病理类型（*χ*^2^=0.414, *P*=0.520）、p-TNM分期（*χ*^2^=0.068, *P*=0.794）及吸烟史（*χ*^2^=0.206, *P*=0.650）无明显关系；生存分析显示miRNA-221高表达组患者生存期显著低于低表达组（*P* < 0.001）。*Cox*风险比例模型分析显示miRNA-221表达可作为非小细胞肺癌患者预后不良的标志。

**结论:**

miRNA-221的高表达提示非小细胞肺癌患者预后较差，是潜在的肺癌预后预测分子标志物。

## Abstract (en)

**Background and objective:**

MicroRNAs were important regulators of tumors and the expression of miRNA-221 was associated with malignant proliferation and invasion in many tumors. The aim of this study is to explore the expression of miRNA-221 in non-small cell lung cancer (NSCLC) tissues and its correlation with prognosis.

**Methods:**

We retrospectively analyzed the clinical characteristics of 117 NSCLC patients who underwent surgery in our hospital from November 2005 to January 2007. Real-time PCR was used to detect the expression of miRNA-221. *Chi-square* test was utilized to analyze the relationship between miRNA-221 expression and clinicopathologic features. Survival curves were plotted by using the *Kaplan-Meier* method and compared by the *Log-rank* test. A *Cox* proportional hazard regression model was applied to examine the joint effect of covariants. A *P*-value less than 0.05 was evaluated as statistically significant.

**Results:**

The patients were divided into two groups according to the relatively expression of miRNA-221. No statistically significance was observed between the expression of miRNA-221 and the clinicopathologic parameters, including gender (*χ*^2^=0.070, *P*=0.791), histology (*χ*^2^=0.414, *P*=0.520), p-TNM stage (*χ*^2^=0.068, *P*=0.794) and history of smoking (*χ*^2^=0.206, *P*=0.650). Kaplan-Meier analysis showed that high expression of miRNA-221 was closely associated with a shorter survival time (*P* < 0.001). Finally, both univariate and multivariate analyses demonstrated that high miRNA-221 expression might be a poor prognostic marker of NSCLC patients.

**Conclusion:**

Our results indicated that down-regulation of miRNA-221 was associated with poor prognosis of patients with NSCLC. MiRNA-221 may serve as a molecular prognosis marker for patients with NSCLC.

微小RNAs（microRNAs, miRNAs）是一类长度为20个-24个核苷酸的小的内源性非编码调控RNA，通过靶向结合mRNA的3’非翻译区（3′-UTR）导致mRNA的降解或翻译抑制^[1, 2]^，从而调控靶基因表达，参与细胞增殖、分化、凋亡等一系列重要生物学进程。肿瘤的发展涉及多种基因在转录及转录后、翻译及翻译后等进程，而这些进程受到不同的因子调控^[3]^。miRNA就是其中一类重要的调控因子^[4]^，通过调节关键的癌基因或抑癌因子，miRNAs与肿瘤的发生、进展等密切相关^[5]^。目前已发现的miRNAs约有700多种，miRNA-221是其中一个重要的miRNA分子。研究^[6]^表明miRNA-221在包括肺癌在内的多种肿瘤中表达明显上调，提示miRNA-221可能是一种潜在的致癌miRNA。近年来，研究者对于miRNA-221在促进肿瘤细胞增殖迁移等方面研究相对广泛，然而其在肺癌预后方面的研究很少。本研究回顾性分析江苏省肿瘤医院117例非小细胞肺癌（non-small cell lung cancer, NSCLC）患者的临床资料，并对上述患者肺癌组织标本进行miRNA-221表达检测，分析生存期与该基因表达之间的关系，试图探讨miRNA-221作为NSCLC预后分子标志的可能性。

## 1 资料与方法

### 1.1 标本来源

收集江苏省肿瘤医院2005年11月-2007年1月期间手术切除并经病理确诊为NSCLC的石蜡标本117例。所有患者术前均未接受过放化疗。肺癌病理分型及分期标准按照1992年UICC-AJCC（Union for International Cancer Control-American Joint Committee on Cancer）对NSCLC的诊断标准执行。所有患者都签署了知情同意书。本研究经江苏省肿瘤医院医学伦理委员会批准。

### 1.2 主要试剂及仪器

高速冷冻离心机；NanoDropND-1000紫外分光度计（NanoDrop）；7300 Real-time PCR System(Applied Biosystems公司)、9700 Thermocycler反应仪；Recover All Total Nucleic Acid Isolation Kit（Applied Biosystems公司）；has-miRNA-221引物、内参RNU6B引物（RiboBio公司）；Real-time逆转录试剂盒（RR037A）（TakaRa公司）；SYBR® Select Master Mix试剂盒（Applied Biosystems公司）。

### 1.3 实时定量PCR方法

#### 1.3.1 蜡块组织总RNA提取

利用Recover All Total Nucleic Acid Isolation Kit（Applied Biosystem）试剂盒提取肺癌石蜡组织总RNA, 严格遵照说明书进行各操作步骤。总RNA用50 μL DEPC水稀释，储存于-80 ℃备用。采用NanoDropND-1000紫外分光度计测量总RNA OD260/280。

#### 1.3.2 逆转录反应

按照逆转录试剂盒操作程序进行逆转录反应，RNA的量为0.5 μg，冰上加入下列试剂：总RNA 1 μL，5×PrimeScript Buffer 5 mL，PrimeScript RT Enzyme Mix I 1 μL，stem-loop RT primer 2 μL，DEPC水，总体积20 μL。所有试剂加入后，42 ℃反应15 min、85 ℃ 5 s、4 ℃ 15 min，后置-80 ℃保存备用。

#### 1.3.3 实时定量PCR

实时定量PCR反应体系如下：SYBR^®^Green Realtime PCR Master Mix 10 μL，上游引物（5 μM）1 μL，下游引物（5 μM）1 μL，cDNA 2 μL，加DEPC水至20 μL。将反应管置7300 Real-time PCR反应仪（Applied Biosystem）中，反应条件为95 ℃预变性10 min后，按下述条件扩增40个循环：95 ℃ 15 s，60 ℃ 1 min。实验重复三次。

### 1.4 随访

所有117例患者进行电话和书信形式随访，了解生存情况等信息，随访截止日期为2011年3月，中位随访时间40个月（范围3个月-64个月）。

### 1.5 统计学方法

MiRNA-221相对表达量采用2^-△△Ct^方法^[7]^，△△Ct = △Ct – Avg.△Ct = (CtmiRNA-221 – CtU6) –Avg.(CtmiRNA-221 – CtU6)。把2^-△△Ct^中位数作为分界值，将患者分为miRNA-221低表达组和高表达组。MiRNA-221表达与临床病理学参数统计方法采用*χ*^2^检验；采用Kaplan-Meier方法进行生存分析，并采用*Log-rank*检验比较两组生存率；采用*Cox*风险比例模型进行各个协变量的联合效应分析。应用SPSS 21.0统计软件进行统计学处理。*P* < 0.05为差异有统计学意义。

## 2 结果

### 2.1 患者基本资料

在117例NSCLC患者中，男性86例，女31例。中位年龄60岁（范围：41岁-79岁）。病理证实为腺癌72例，鳞癌45例。病理分期Ⅰ期41例，Ⅱ期-Ⅲa期76例。其中44例无吸烟史，73例有吸烟史。具体见表 1。

**1:** miRNA-221表达与NSCLC患者临床病理参数的关系 Correlation of miRNA-221 expression and the clinical characteristics of 117 NSCLC patients

Characteristic	*n*	miRNA-221 expression		*χ*^2^	*P*
		High [*n* (%)]	Low [*n* (%)]		
Age (yr)				4.547	0.033
≥60	61	25 (41.0)	36 (59.0)		
60	56	34 (60.7)	22 (39.3)		
Gender				0.070	0.791
Male	86	44 (51.2)	42 (48.8)		
Female	31	15 (48.4)	16 (51.6)		
Histology				0.414	0.520
SCC	45	21 (46.7)	24 (53.3)		
ADC	72	38 (52.8)	34 (47.2)		
Stage p-TNM				0.068	0.794
Ⅰ	41	20 (48.8)	21 (51.2)		
Ⅱ-Ⅲa	76	39 (51.3)	37 (48.7)		
Smoke				0.206	0.650
No	44	21 (47.7)	23 (52.3)		
Yes	73	38 (52.1)	35 (47.9)		
SCC: squamous cell carcinoma; ADC: adenosquamous carcinoma; NSCLC: non-small cell lung cancer.					

### 2.2 miRNA-221表达与NSCLC患者临床资料关系

根据miRNA-221的表达，肺癌患者被相对分为miRNA-211高表达和低表达组。117例NSCLC组织中，59例miR-211相对高表达。统计结果显示，miRNA-221的表达量与患者年龄（*χ*^2^=4.547, *P*=0.033）相关。其中60岁以上肺癌患者组织中有25例miRNA-221呈高表达，36例低表达；60岁以下患者中，miRNA-221呈高表达34例，低表达22例。miRNA-221的表达量与性别（*χ*^2^=0.070, *P*=0.791）、病理类型（*χ*^2^=0.414, *P*=0.520）、p-TNM分期（*χ*^2^=0.068, *P*=0.794）及吸烟史（*χ*^2^=0.206, *P*=0.650）均无统计学意义（*P* > 0.05）（表 1）。

### 2.3 miRNA-221表达与NSCLC患者生存时间的关系

利用*Kaplan-Meier*对117例患者进行生存分析，结果显示：miR-211高表达组患者中位生存时间35.34个月，较miRNA-221低表达组患者（平均48.72个月）明显缩短，两组生存期差异有统计学意义（*Log-rank*检验：*P*=0.006）（图 1）。*Cox*风险比例模型单因素分析显示：肺癌p-TNM分期偏晚（*P*=0.003）和miR-211高表达（*P*=0.007）与NSCLC患者预后相关（表 2）。根据*Cox*回归模型多因素分析的结果，p-TNM分期偏晚（*P*=0.004）和miR-211高表达（*P*=0.005）是NSCLC患者预后的独立危险因素（表 2）。

**1:**
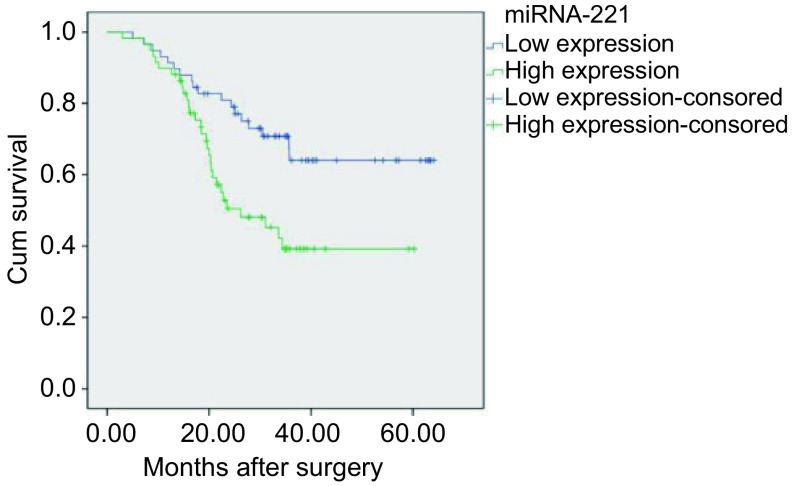
不同miRNA-221表达水平NSCLC患者的生存分析 Survival analysis of different miRNA-221 expression in NSCLC patients

**2:** NSCLC患者总生存期单因素及多因素*Cox*回归分析 Univariate and multivariate analyses of overall survival (OS) of 117 NSCLC patients

Characteristic	Univariate analysis			Mutivariate analysis	
	HR (95%CI)	*P*		HR (95%CI)	*P*
Gender (Female *vs* Male)	0.775 (0.395-1.521)	0.459		1.042 (0.403-2.692)	0.932
Age (≥60 *vs* < 60）	1.09 (0.618-1.924)	0.766		1.11 (0.592-2.083)	0.745
Histology (ADC *vs* SCC)	0.84 (0.47-1.499)	0.555		0.755 (0.39-1.461)	0.404
p-TNM stage（Ⅱ-Ⅲa *vs* Ⅰ）	2.961 (1.433-6.118)	0.003		2.929 (1.399-6.132)	0.004
Smoke (Yes vs No)	1.016 (0.57-1.812)	0.957		0.787 (0.349-1.772)	0.563
miR221 expression (High vs Low)	2.247 (1.246-4.051)	0.007		2.425 (1.314-4.475)	0.005

## 3 讨论

NSCLC主要包括腺癌和鳞状细胞癌，约占据全球因癌症死亡患者的1/6^[8]^。尽管在临床与实验肿瘤学方面均取得很多进展，但肺癌的预后仍然不佳，5年总体生存率大约11%^[9]^。因此，了解详细的机制对于诊断、预防和治疗NSCLC是必不可少的。最近，越来越多的证据^[10]^表明，miRNAs参与NSCLC的发病机制；miRNAs可作为一项工具来调控与肿瘤发生发展相关的信号通路和一些关键基因或因子，与肿瘤分化、发生、侵润和转移等多个环节密切相关。而miRNA-221是miRNAs大家族的重要一员，位于人类X染色体p11.3。文献表明miRNA-221在脑胶质瘤^[11]^、甲状腺乳头状癌^[12]^、乳腺癌^[13]^、肝细胞癌^[14]^、前列腺癌^[15]^和卵巢癌^[16]^等多种癌症中高表达，在肿瘤发生过程中发挥类似于致癌基因的作用。研究发现在这些肿瘤中，过表达的miRNA-221可以靶向作用于重要的肿瘤抑制基因如*P27^/Kip1^*、*P57*、*Puma*、*ER-α*、*FOXO3*、*PTEN*、*TIMP3*、*DDIT4*、*Bim*，继而激活细胞周期和AKT旁路或阻断TNF相关凋亡诱导配体（TRAIL），诱导肿瘤增殖和侵袭；而在舌鳞癌和甲状腺癌中，miRNA-221也可以作用c-Kit或MMP1抑制肿瘤增殖，起着抑癌miRNA的作用^[6]^。此外Garofalo等^[17, 18]^研究发现，肺癌中过表达的miRNA211可以靶向结合抑癌基因PTEN和TIMP3，通过激活AKT旁路和金属钛酶从而诱导TRAIL抵抗和促进肺癌细胞的增殖、迁移和浸润。这些发现提示了miRNA-221在肺癌中可能起着关键的作用。

本研究回顾分析了我院117例NSCLC患者的临床资料，并采用RT-PCR方法对上述肺癌患者组织标本中miRNA-221的表达水平进行检测，应用*Kaplan-Meier*法研究miRNA-221在NSCLC预后方面的意义。结果发现，117例NSCLC患者的临床病理资料中，miRNA-221表达与性别、病理类型及分期、吸烟史等无明显相关；卡方分析提示miRNA-221表达量与患者年龄相关，而*Cox*单变量和多变量分析表明总生存期与年龄无关，可以认为miRNA-211表达与年龄相关没有临床意义。*Cox*风险比例模型单因素分析显示miRNA-221高表达与NSCLC患者预后呈明显相关，多因素分析表明miR-221高表达（*P*=0.005）是NSCLC患者预后的独立危险因素。而生存分析提示miRNA-221表达与患者总生存期呈负相关，即微小RNA-221表达水平高的患者生存时间较短。以上结果表明miRNA-221可能是NSCLC预后的一个重要指标。

本研究为回顾性研究，临床资料不够完善，例如缺少瘤旁组织和肿瘤组织的对照。对于肺癌患者的分组也可以更加地严格和精确，比如针对更细致的病理分期或分化程度进行分组。

综上所述，miRNA-221高表达提示NSCLC预后较差，miRNA-221可作为NSCLC预后的一个重要分子标志。未来值得进一步开展严格的前瞻性临床研究进一步证实该结果的可靠性。
